# Combination of *Helicobacter pylori* Antibody and Serum Pepsinogen as a Good Predictive Tool of Gastric Cancer Incidence: 20-Year Prospective Data From the Hisayama Study

**DOI:** 10.2188/jea.JE20150258

**Published:** 2016-12-05

**Authors:** Fumie Ikeda, Kentaro Shikata, Jun Hata, Masayo Fukuhara, Yoichiro Hirakawa, Tomoyuki Ohara, Naoko Mukai, Masaharu Nagata, Daigo Yoshida, Koji Yonemoto, Motohiro Esaki, Takanari Kitazono, Yutaka Kiyohara, Toshiharu Ninomiya

**Affiliations:** 1 Department of Environmental Medicine, Graduate School of Medical Sciences, Kyushu University, Fukuoka, Japan; 1 九州大学大学院医学研究院 環境医学分野; 2 Department of Medicine and Clinical Science, Graduate School of Medical Sciences, Kyushu University, Fukuoka, Japan; 2 九州大学大学院医学研究院 病態機能内科学; 3 Center for Cohort Studies, Graduate School of Medical Sciences, Kyushu University, Fukuoka, Japan; 3 九州大学大学院医学研究院 総合コホートセンター; 4 Department of Neuropsychiatry, Graduate School of Medical Sciences, Kyushu University, Fukuoka, Japan; 4 九州大学大学院医学研究院 精神病態医学; 5 Biostatistics Center, Kurume University, Fukuoka, Japan; 5 久留米大学 バイオ統計センター

**Keywords:** *Helicobacter pylori*, pepsinogen, prospective studies, stomach neoplasms, biological markers, ヘリコバクター・ピロリ, ペプシノゲン, 前向き追跡研究, 胃癌, 生物学的指標

## Abstract

**Background:**

There is little information regarding whether the combination of *Helicobacter pylori* (*H. pylori*) antibody and serum pepsinogen (sPG), which is a marker of the degree of atrophic gastritis, has a discriminatory ability for detecting incident gastric cancer. We examined this issue in a long-term prospective cohort study of a Japanese population.

**Methods:**

A total of 2446 Japanese community-dwelling individuals aged ≥40 years were stratified into four groups according to baseline *H. pylori* serological status and sPG: Group A (*H. pylori*[−], sPG[−]), Group B (*H. pylori*[+], sPG[−]), Group C (*H. pylori*[+], sPG[+]), and Group D (*H. pylori*[−], sPG[+]), and participants were followed up prospectively for 20 years.

**Results:**

During the follow-up, 123 subjects developed gastric cancer. Compared with that in Group A, the cumulative incidence of gastric cancer was significantly increased in Groups B, C, and D, whereas no significant difference was found between Groups C and D. The multivariable-adjusted risk of gastric cancer was significantly increased in Group B (hazard ratio [HR], 4.08; 95% confidence interval [CI], 1.62–10.28) and in Groups C and D combined (HR 11.1; 95% CI, 4.45–27.46). When the multivariable model with *H. pylori* antibody was changed into that with the combination of *H. pylori* antibody and sPG, the C statistics for developing gastric cancer increased significantly (0.773 vs 0.732, *P* = 0.005), and the continuous net reclassification improvement value was 0.591 (*P* < 0.001).

**Conclusions:**

Our findings suggest that the combination of *H. pylori* antibody and sPG is a useful tool for predicting the development of gastric cancer.

## Abstract (ja)

**背景：:**

萎縮性胃炎の程度の指標であるヘリコバクター・ピロリ（*Helicobacter pylori*：*H.pylori*）抗体価とペプシノゲン(sPG)検査を組み合わせた検査の、将来の胃癌罹患予測における有用性は、ほとんど検討されていない。そこで、わが国の地域住民を対象とした長期間の前向きコホート研究の成績を用いて、この問題について検討した。

**方法：:**

40歳以上の日本人地域住民2,446人を追跡開始時の*H.pylori*抗体陽性の有無とsPG検査の陽性の有無の組み合わせにより4群：A群(*H.pylori*[-] sPG[-]）、B群(*H.pylori*[+]sPG[-])、C群(*H.pylori*[+]sPG[+])、D群(*H.pylori*[-]sPG[+])に分類し、前向きに20年間追跡した。

**結果：:**

追跡期間中に123人が胃癌を発症した。胃癌の累積罹患率は、A群に比べB、C、D群で有意に上昇したが、C群とD群の間に有意差は認められなかった。多変量調整後の胃癌罹患のハザード比は、B群　4.08(95％信頼区間1.62-10.28)、C+D群　11.1(95％信頼区間4.45-27.46)であった。*H.pylori*抗体価と胃癌の他の危険因子で構成された胃癌罹患の予測モデルを*H.pylori*抗体およびsPG検査の組み合わせと胃癌の他の危険因子で構成された予測モデルに変更したところ、予測モデルのC統計量は0.732から0.773と有意に上昇した（*P*=0.005）。また、Continuous net reclassification improvement(cNRI)も、2つの予測モデルの差が0.591と有意に胃癌罹患予測能は改善した（*P*<0.001）。

**結論：:**

*H.pylori*抗体価とsPG検査を組み合わせて用いることは、将来の胃癌罹患リスクを予測する上で有用な手段である。

## INTRODUCTION

*Helicobacter pylori* (*H. pylori*), which was discovered in 1983, is known to be a strong and convincing risk factor for the development of gastric cancer.^1^^–^^4^ Long-term *H. pylori* infection causes chronic atrophic gastritis and gastric mucosal atrophy, which have been recognized as precursors of gastric cancer based on clinicopathologic and epidemiologic studies.^5^^–^^8^ Both *H. pylori* infection and chronic atrophic gastritis have been detected with blood tests by assaying serum anti-*H. pylori* IgG^9^ and serum pepsinogen (sPG) levels,^10^^,^^11^ and it has been reported that each of these conditions was a predictive marker for the development of gastric cancer.^2^^,^^12^^–^^15^ However, it is known that antibody to *H. pylori* is diminished due to severe atrophic gastritis,^16^ and tests for sPG are negative when atrophic gastritis does not occur despite the existence of *H. pylori* infection.^17^ In order to cover the limitations of *H. pylori* antibody and sPG, the combination of *H. pylori* antibody and sPG, which is called the ABC method in Japan, was proposed,^17^ and this method is considered to represent the degree of chronic gastritis more accurately. In Japan, the combination of *H. pylori* antibody and sPG has started to be applied to mass screening for gastric cancer.^17^ Although some prospective studies have shown that the combination of *H. pylori* antibody and sPG was significantly associated with the development of gastric cancer,^18^^–^^21^ the follow-up period of these studies was relatively short (generally fewer than 15 years), and no study has examined whether this method serves as a more useful tool for predicting gastric cancer than the measurement of *H. pylori* antibody alone.

The purposes of the present study were to conduct a long-term (20-year) prospective investigation of the relationship of the combination of *H. pylori* antibody and sPG with gastric cancer occurrence, and to investigate the discriminatory ability of this method to identify subjects who are at increased risk of developing gastric cancer in a general Japanese population.

## METHODS

### Study population

A population-based prospective study has been underway since 1961 in the town of Hisayama, a suburb of Fukuoka City on Kyushu Island, Japan. A detailed description of this survey was published previously.^2^^,^^22^ In 1988, of a total 3390 residents aged ≥40 years on the town registry, of whom 2742 consented to participate in the examination (participation rate: 80.9%) and underwent a comprehensive assessment, including an interview covering medical history and the measurement of *H. pylori* antibody and sPG levels. After excluding 130 individuals with a prior history of gastrectomy or gastric cancer, 161 individuals in whom *H. pylori* antibody or sPG levels were not measured, and 5 individuals who died during the screening period, a total of 2446 subjects (1016 men and 1430 women; mean age, 58.3 years) were enrolled in the study.

### Follow-up survey

The subjects were followed prospectively for 20 years, from December 1988 to November 2008, using repeated health examinations or a daily monitoring system established by the study team and local physicians or members of the Health and Welfare Office of the town. Health status was checked once yearly by mail or telephone for any subjects who did not undergo a regular examination or who moved out of town.^22^ All participants were followed up completely over the 20 years. The cases of gastric cancer were surveyed in local clinics in the town and hospitals around the town using medical records of barium radiographic and/or upper endoscopic examinations, including biopsy diagnosis.^2^ We also checked all records from annual mass screenings for gastric cancer that applied upper gastro-intestinal series.^2^ Further, to find any concealed gastric cancer, an autopsy was performed on 554 (71.4%) of the 776 subjects who died during the follow-up period. The diagnosis of all cases of gastric cancer was confirmed using histological examination of tissues obtained in surgery, including gastrectomy, endoscopic mucosal resection, endoscopic submucosal dissection, and autopsy. Pathologic diagnosis and classification of identified gastric cancers were made according to the guidelines proposed by the Japanese Gastric Cancer Association.^23^ During the follow-up, gastric cancer developed in 123 subjects (86 men and 37 women). There were 3 concealed cases (2.4%), which were first confirmed through autopsy.

### Laboratory testing and risk factor measurements

At baseline, a serum sample was collected from each subject and was stored at −20°C until the assay for serum IgG antibodies to *H. pylori* and sPG I and II. Serum *H. pylori* antibodies were assayed through quantitative enzyme immunoassay using a commercial kit (HM-CAP; Enteric Products Inc., Westbury, NY, USA) in 1997. Assay values were interpreted as either positive or negative based on the manufacturer’s instructions. The measurement of sPG concentrations was carried out through immunoradiometric assay (PG I/II RIA BEAD; Dinabot Co. Ltd., Tokyo, Japan), a modified method of radioimmunoassay, in 2002. On the basis of the sPG test proposed by Miki et al,^10^ which is a widely-accepted method in Japan, the study participants were classified into two groups, those with a positive sPG (sPG I levels of ≤70 ng/mL and PG I/II ratios of ≤3.0) and those with a negative sPG.

To assess the independent relationship of risk stratification using the combination of *H. pylori* antibody and sPG with gastric cancer occurrence, several baseline factors, in addition to age and sex, were gathered. Information about history of peptic ulcer disease, family history of cancer, alcohol intake, and smoking habits was obtained using a questionnaire administered to each subject, and alcohol intake and smoking habits were categorized as in current use or not. Height and weight were measured with the subject in light clothes without shoes, and body mass index (kg/m^2^) was calculated. Hemoglobin A1c was measured using high-performance liquid chromatography. Serum cholesterol levels were determined using an enzymatic autoanalyzer. Data on dietary factors were obtained through the semiquantitative food frequency method, which was validated in a prior study.^24^ The daily nutrient intakes were calculated using the 4th revision of the Standard Tables of Food Consumption in Japan,^25^ and the nutritional element intakes were adjusted for energy intake using the method of Willet and Stampfer.^26^

### Statistical analysis

According to *H. pylori* serological status and sPG levels at baseline, we classified all of the subjects into four groups: Group A, negative for *H. pylori* antibodies and sPG; Group B, positive for *H. pylori* antibodies and negative for sPG; Group C, positive for *H. pylori* antibodies and sPG; and Group D, negative for *H. pylori* antibodies and positive for sPG. The cumulative and age- and sex-adjusted incidences of gastric cancer were calculated using the Kaplan-Meier method and the person-year method, respectively. The difference in cumulative incidence was examined using a log rank test. The age- and sex-adjusted and multivariable-adjusted hazard ratios (HRs) and their 95% confidence intervals (CIs) were estimated using a Cox proportional hazards model. In the multivariable analysis, we selected clinically or biologically plausible risk factors for gastric cancer, which are listed in Table 1.^27^^–^^32^ Of these risk factors, hemoglobin A1c, total cholesterol, smoking habit, and dietary salt and vitamin A intakes were independent risk factors for gastric cancer in our previous cohort studies.^28^^–^^32^ Accordingly, we considered that other vitamins and dietary fiber, which were taken together with vitamin A, were potential confounding factors and included them in the model. We then performed backward selection with a value of *P* < 0.2 being required for a factor to remain in the model. As a result, age, sex, body mass index, total cholesterol, hemoglobin A1c, smoking habits, daily total energy, and salt intake made up the final list of confounding factors used for multivariable adjustment, and the model composed of these selected variables was defined as the basic model. The goodness of fit between observed and predicted number of incident gastric cancer in the basic model was tested using the Hosmer-Lemeshow test.^33^ To compare the accuracy of risk assessment for the occurrence of gastric cancer between the basic model with and without *H. pylori* antibody, and between the basic model with *H. pylori* antibody and those with the combination of *H. pylori* antibody and sPG, we examined C statistics analogous to the area under the receiver operating curve.^34^ The statistical significance of differences in this analysis was compared using the method of DeLong et al.^35^ Moreover, the increased discriminatory values of the *H. pylori* antibody and the combination of *H. pylori* antibody and sPG were examined using net reclassification improvement (NRI) and integrated discrimination improvement (IDI).^36^ The NRI evaluates the difference in estimated predicted probabilities between different models in individuals with and without events. When the predicted probabilities using the new model shift to be higher than the old model in the individuals with events and the opposite is also shown in the individuals without events, the new model may have more accurate discriminatory ability for the target event. In this analysis, we classified the probability of the risk of gastric cancer for 20 years into three categories of <3.0%, 3.0% to 12.0%, and >12.0%, because the median value of the predicted probabilities was 3.3% in subjects without occurrence of gastric cancer and 12.1% in those with occurrence of gastric cancer. The continuous NRI value was also estimated using the predicted probabilities taken as continuous variables. The IDI evaluates the difference in the average of the estimated predicted probabilities for subjects with and without events between different models. The proportion of missing values was 2.5% for all the variables included in the model. A two-sided *P* value <0.05 was considered statistically significant in all analyses. Statistical analyses were conducted using Statistical Analysis Software (SAS) version 9.3 (SAS Institute, Cary, NC, USA).

**Table 1.** Baseline characteristics of the subgroups classified according to the combination of *Helicobacter pylori* antibody and serum pepsinogen

	Group				

	A	B	C	D	
*Helicobacter pylori* antibody status	Negative	Positive	Positive	Negative	
Pepsinogen status	Normal	Normal	Atrophic	Atrophic	Total
Subjects, *n*	606	1126	635	79	2446
Men, %	34.7	46.6	40.6	29.1	41.5
Age, years	57.9 (11.9)	56.4 (10.7)	61.6 (11.2)	62.1 (11.0)	58.3 (11.4)
Pepsinogen I, ng/mL	48.6 (38.1, 64.6)	68.5 (52.5, 87.1)	38.8 (24.9, 54.0)	31.3 (14.1, 50.5)	54.5 (38.5, 71.9)
Pepsinogen II, ng/mL	6.9 (5.4, 9.9)	16.6 (11.5, 25.8)	18.4 (13.6, 23.4)	17.2 (11.1, 20.9)	14.8 (8.8, 22.2)
Pepsinogen I/II, ratio	6.9 (5.6, 8.0)	3.9 (3.2, 4.9)	2.1 (1.6, 2.6)	1.9 (1.5, 2.5)	3.6 (2.5, 5.6)
Body mass index, kg/m^2^	23.0 (3.1)	23.2 (3.1)	22.6 (3.0)	23.5 (3.4)	23.0 (3.1)
Hemoglobin A1c, %	5.9 (0.7)	6.0 (0.9)	5.9 (0.8)	6.0 (0.9)	6.0 (0.8)
Total cholesterol, mmol/L	5.48 (1.11)	5.34 (1.04)	5.28 (1.11)	5.30 (1.03)	5.36 (1.08)
Smoking habits, %	21.3	27.0	24.7	13.9	24.6
Alcohol intake, %	28.1	34.3	30.6	22.8	31.4
History of peptic ulcer (%)	9.4	19.7	13.9	7.6	15.3
Family history of cancer, %	8.3	10.1	8.0	10.1	9.1
Total energy intake, KJ/day	6981 (1610)	7262 (1830)	7142 (1699)	6854 (1768)	7149 (1747)
Total fat intake, g/day	49.6 (11.0)	48.4 (10.1)	47.1 (10.7)	48.2 (10.1)	48.4 (10.5)
Salt intake, g/day	13.1 (4.5)	13.1 (4.6)	13.2 (5.1)	12.7 (4.7)	13.1 (4.7)
Vitamin A intake, µg RE/day	880.8 (332.3)	856.9 (334.2)	869.2 (343.3)	915.9 (309.6)	867.8 (335.3)
Vitamin B1 intake, mg/day	0.83 (0.42)	0.80 (0.40)	0.80 (0.37)	0.85 (0.42)	0.81 (0.40)
Vitamin B2 intake, mg/day	1.18 (0.31)	1.16 (0.31)	1.12 (0.32)	1.17 (0.31)	1.15 (0.31)
Vitamin C intake, mg/day	78.2 (32.5)	75.4 (33.3)	78.2 (35.1)	85.3 (30.0)	77.1 (33.5)
Dietary fiber intake, g/day	11.3 (3.7)	10.7 (3.4)	10.5 (3.3)	11.5 (3.3)	10.8 (3.4)

Continuous variables are expressed as the mean (standard deviation) or median (interquartile range).

Groups A to D, see text for details.

### Ethical considerations

This study protocol was approved by the Kyushu University Institutional Review Board for Clinical Research, and written informed consent for medical research was obtained from the study subjects.

## RESULTS

The baseline clinical characteristics of the study population according to the combination of *H. pylori* antibody and sPG are summarized in Table 1. Among the 2446 participants, 606 (24.8%) were categorized as Group A, 1126 (46.0%) as Group B, 635 (26.0%) as Group C, and 79 (3.2%) as Group D subjects. The mean age of each group tended to increase and the mean value of total cholesterol levels tended to decrease in order from Group A to D.

The cumulative incidence of gastric cancer by group is shown in Figure. Groups B, C, and D had significantly higher incidence of gastric cancer than Group A (*P* < 0.01 for all), but no significant difference in incidence was found between Groups C and D (*P* = 0.53). Therefore, we combined subjects in Groups C and D for the following analysis. The age- and sex-adjusted incidence and HR of gastric cancer increased significantly from groups A to C and D combined (Table 2). This relationship remained substantially unchanged even after adjustment for the following confounding factors: age, sex, body mass index, total cholesterol, hemoglobin A1c, smoking habits, and total energy and salt intakes. The multivariable-adjusted HR of gastric cancer was 4.08 (95% CI, 1.62–10.28; *P* = 0.003) in Group B and 11.1 (95% CI, 4.45–27.46; *P* < 0.001) in Groups C and D combined.

**Figure.**
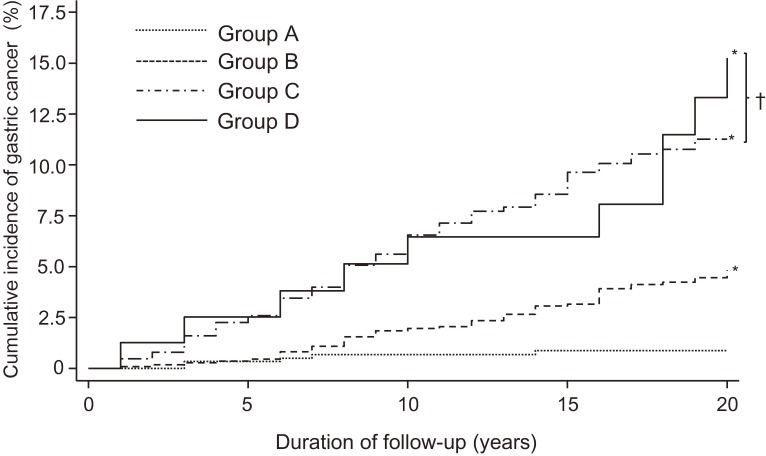
20-year cumulative incidence of gastric cancer according to the combination of *H. pylori* antibody and serum pepsinogen at baseline. Groups A to D, see text for details. *P < 0.01 vs Group A, †*P* = 0.53 by log rank test.

**Table 2.** Adjusted incidence rate and hazard ratio with 95% confidence interval for gastric cancer according to the combination of *Helicobacter pylori* antibody and serum pepsinogen

	Group		

	A	B	C+D
Subjects, *n*	606	1126	714
Cases, *n*	5	48	70
Age- and sex-adjusted incidence rate, /1000 person-years	0.6	2.4	6.7
Age- and sex-adjusted HR, 95% CI	1.00	4.43 (1.76–11.14)^a^	11.6 (4.68–28.87)^a^
Multivariable-adjusted HR, 95% CI	1.00	4.08 (1.62–10.28)^a^	11.1 (4.45–27.46)^a^

HR, hazard ratio; CI, confidence interval.

Multivariable-adjustment was made for age, sex, body mass index, total cholesterol, hemoglobin A1c, smoking habits, and daily total energy and salt intake. Groups A to D, see text for details.

^a^*P* < 0.01 vs Group A.

Finally, we evaluated whether the combination of *H. pylori* antibody and sPG improves the accuracy of gastric cancer risk assessment. The basic model including age, sex, body mass index, total cholesterol, hemoglobin A1c, smoking habits, and daily total energy and salt intakes had good discrimination (c = 0.714) and calibration (Hosmer-Lemeshow test, χ^2^ = 8.03 [degrees of freedom = 8], *P* = 0.43). Next, we compared the discriminatory abilities of the basic model with and without *H. pylori* antibody. The C statistic significantly increased from 0.714 to 0.732 (*P* = 0.049). Significant improvements were also found in the NRI with three categories (0.090, Z_NRI_ = 2.34, *P* = 0.02, eTable 1), continuous NRI (0.330, Z_NRI_ = 3.56, *P* < 0.001), and IDI (0.006, Z_IDI_ = 2.10, *P* = 0.04). We then compared the discriminatory abilities of the basic model with *H. pylori* antibody alone and with the combination of *H. pylori* antibody and sPG. The C statistics increased significantly when changing the former model to the latter (from 0.732 to 0.773, *P* = 0.005). Cross-tabulation of the predicted probabilities of gastric cancer occurrence for 20 years estimated using the model is shown in Table 3. After altering the model with *H. pylori* antibody to that with the combination of *H. pylori* antibody and sPG, the three category NRI and continuous NRI values were 0.153 (Z_NRI_ = 2.76, *P* = 0.006) and 0.591 (Z_NRI_ = 6.38, *P* < 0.001), respectively. Likewise, the IDI also significantly improved (IDI = 0.038, Z_IDI_ = 4.56, *P* < 0.001). The sensitivity analysis accounting for competing risk of death did not make any material difference in the findings (eTable 2).

**Table 3.** Reclassification for 20-year predicted absolute risk of gastric cancer development

Number of subjects who developed gastric cancer				
	Basic model with the combination of *H. pylori* antibody and sPG			
Basic model with *H. pylori* antibody	<3.0%	3.0%–12.0%	>12.0%	Total

<3.0%	8	11	0	19
3.0%–12.0%	9	25	17	51
>12.0%	0	8	45	53

Total	17	44	62	123


Number of subjects who did not develop gastric cancer				
	Basic model with the combination of *H. pylori* antibody and sPG			
Basic model with *H. pylori* antibody	<3.0%	3.0%–12.0%	>12.0%	Total

<3.0%	762	158	3	923
3.0%–12.0%	298	623	105	1026
>12.0%	0	114	200	314

Total	1060	895	308	2263

*H. pylori*, *Helicobacter pylori*; sPG, serum pepsinogen.

The basic model included age, sex, body mass index, total cholesterol, hemoglobin A1c, smoking habits, and daily total energy and salt intakes.

## DISCUSSION

In the present study, we demonstrated that high-risk status based on risk stratification using the combination of *H. pylori* antibody and sPG was significantly associated with the occurrence of gastric cancer. This relationship remained significant even after adjusting for other confounding factors. To the best of our knowledge, this is the first epidemiologic study to investigate whether this method further improves discriminatory ability for predicting future gastric cancer over a lengthy period compared with *H. pylori* antibody alone. These findings highlight the clinical usefulness of this method in the identifying a high risk group for gastric cancer within a general population.

The combination of *H. pylori* antibody and sPG has been considered to reflect the degree of health of the stomach, with Group A subjects being free of *H. pylori* infection and atrophic gastritis, Group B subjects being infected with *H. pylori* but free of atrophic gastritis, Group C subjects having both *H. pylori* infection and atrophic gastritis, and Group D subjects exhibiting a loss of *H. pylori* presumably due to advanced gastric atrophy. In our study, the subjects of Groups B, C, and D had significantly higher risk for the development of gastric cancer compared with those of Group A, and the risk of cancer steadily increased from Group A to D. There have been three published studies from Japan^18^^–^^20^ and a study from China^21^ that prospectively examined the relationship of the combination of *H. pylori* antibody and sPG with the risk of gastric cancer, and we found similar results to other Japanese studies.^18^^–^^20^ Most of the other prospective studies followed subjects for less than 15 years, whereas the follow-up period of our study was the longest, at 20 years. Our present findings, taken together with these others, imply that positive tests for *H. pylori* antibody or sPG are significant long-term markers for the development of gastric cancer.

In recent years, when a new biomarker or model is assessed as a novel risk factor or model, it is commonly compared with an existing marker or model.^37^^,^^38^ To date, however, no studies have examined the predictive ability of the combined use of *H. pylori* antibody and sPG for gastric cancer development. In this study, we assessed this issue using three statistical methods—the C statistic, IDI, and NRI—and all of them demonstrated a significant improvement of the predictive ability of this method for the future risk of gastric cancer. Therefore, our results enhance the evidence that the combination of *H. pylori* antibody and sPG is a useful tool for the identification of subjects at increased risk of future gastric cancer.

In the present study, we found no significant difference in the cumulative incidence of gastric cancer between Groups C and D. A similar finding was observed in a recent meta-analysis.^39^ These results infer that the risk of gastric cancer development in subjects with atrophic gastritis is similar with or without *H. pylori* seropositivity. In addition, the prevalence of Group D subjects in our cohort was quite small (only 3%), and the same was true in other population-based cohort studies. These findings suggest that subjects of Groups C and D can be considered to have almost the same risk of future gastric cancer.

Our subjects in Group B, who had *H. pylori*-induced active inflammation in the stomach without atrophy, had a significant risk of gastric cancer incidence. A study focused on the risk of gastric cancer in subjects with *H. pylori* but without stomach atrophy also clearly demonstrated that subjects with highly active gastritis were at high risk of gastric cancer development, particularly diffuse-type cancer.^40^ Intriguingly, the studies with follow-up period of less than 10 years^18^^–^^20^ failed to reveal a significantly greater risk of gastric cancer in these subjects compared to *H. pylori*- and sPG-negative subjects, whereas a significant increase in cancer risk was observed in the study with follow-up period of more than 10 years,^21^ which is consistent with the results of our study. These findings raise the possibility that subjects with *H. pylori* infection but without stomach atrophy might experience atrophic gastritis through *H. pylori*-induced active inflammation over time and subsequently develop gastric cancer. Therefore, subjects infected with *H. pylori* without atrophic gastritis should also be considered to have moderate risk of gastric cancer.

It is generally believed that most gastric cancer is caused by *H. pylori* infection.^3^ Therefore, it has been debated whether gastric cancer develops in subjects classified into Group A. A prospective study with middle-aged male employees revealed that none of the subjects of Group A developed gastric cancer,^18^ while prospective studies of health check-ups and a population-based cohort reported that gastric cancer did develop in some Group A subjects, though the incidence rate was extremely low.^19^^–^^21^ The latter results were in accordance with ours. Regarding specific features of the gastric cancer developed in subjects without *H. pylori* infection and atrophic gastritis, some studies have reported that cardiac cancer or diffuse-type cancer occurred frequently in this group,^19^^,^^41^ while in such subjects in our cohort (Group A), one cardiac and four distal gastric cancers developed, all of which were intestinal-type. Further clinical and epidemiologic evidence is required to elucidate this issue.

The strengths of our study include its longitudinal population-based design, low selection bias at baseline, perfect follow-up of subjects, and accuracy of diagnosis of gastric cancer, including the use of autopsy findings. Moreover, the follow-up period of this study is much longer than that of other studies, and the relationship of risk stratification using the combination of *H. pylori* antibody and sPG with the development of gastric cancer was examined with consideration for other comprehensive risk factors, whereas other prospective studies did not include this information.^18^^–^^21^ However, some limitations in this study must be mentioned. First, although the use of long-term stored serum is not validated, we used frozen stored serum samples to measure serum IgG antibodies to *H. pylori* and sPG levels. In addition, we used an imported serological kit for *H. pylori*, for which the accuracy of detecting *H. pylori* is limited in Japanese subjects compared with Japanese serological kits.^42^ However, the seroprevalence of *H. pylori* and the mean values of sPG I and II levels in our study were not much different than those in a previous report from Japan.^43^ Moreover, if the misclassification of *H. pylori* positivity and sPG occurred equally among all study subjects, it would have biased our results towards the null hypothesis. Second, although it has been reported that the combination of *H. pylori* antibody and sPG is associated with the prevalence of gastric cancer,^44^ we did not perform a screening survey of the stomach in each subject at baseline using barium radiographic or upper endoscopic examinations. Therefore, we cannot deny the possibility of subclinical gastric cancer at baseline. However, in a study using hospital-based general medical checkups, the prevalence of gastric cancer in healthy subjects was reported to be low (0.19% in the group with *H. pylori* infection and a negative sPG and 0.87% in the group with a positive sPG).^41^ Moreover, in our study, the sensitivity analysis performed after exclusion of subjects who developed gastric cancer during the initial 3-year follow-up period did not show any changes in the results (data not shown). Therefore, we believe that the influence of concealed cancer at the time of recruitment was small enough that it can be neglected. Third, we did not take *H. pylori* eradication therapy into account in our study. In Japan, *H. pylori* eradication became widespread after the year 2000, when the Japanese guideline for treatment of *H. pylori* was established and when such treatment was first covered by the Japanese national health insurance system.^45^ It is reasonable to assume that some subjects would have taken *H. pylori* eradication therapy during the follow-up period, and thus misclassification of categories of *H. pylori* infection status and sPG levels was possible. This would actually lead to underestimation of the risk for the combination of *H. pylori* antibody and sPG, suggesting that the true relationship may be even greater than that found here. Nevertheless, in Japan, the recent widespread use of *H. pylori* eradication therapy is likely to increase chances of misclassification of subjects with atrophic gastritis as being free of *H. pylori* infection and negative for sPG,^46^ and the validity of the combination of *H. pylori* antibody and sPG might decrease in the future. Therefore, the application of the combination of *H. pylori* antibody and sPG to mass screening for gastric cancer should be limited to individuals who have received *H. pylori* eradication therapy. Finally, we could not perform an external validation study for the predictive ability of the combination of *H. pylori* antibody and sPG for gastric cancer. Further large-scale prospective studies will be required to reveal the precise role of this method.

In conclusion, the combination of *H. pylori* antibody and sPG is a significant predictor for the development of gastric cancer over a long-term period. This method is simple, inexpensive, and noninvasive, so it would be highly suitable for identifying high-risk subjects who should receive gastric cancer screening. Further investigations will be required to explore the most cost-effective strategies for mass screening for gastric cancer, especially in Japan and other countries with high morbidity and mortality of gastric cancer.

## ONLINE ONLY MATERIALS

eTable 1. Reclassification of 20-year predicted absolute risk of gastric cancer development.

eTable 2. Reclassification of 20-year predicted absolute risk of gastric cancer development accounting for competing risk of death.

Abstract in Japanese.
