# Supplementary material for: Combination of Helicobacter pylori Antibody and Serum Pepsinogen as a Good Predictive Tool of Gastric Cancer Incidence: 20-Year Prospective Data From the Hisayama Study
Source: J Epidemiol. 2016 Dec 5;26(12):629–36. doi: 10.2188/jea.JE20150258 (PMC5121431; doi:10.2188/jea.JE20150258)
Supplement: eTable 1. [file je-26-629-s001.pdf]

eTable 1. Reclassification for 20-year predicted absolute risk of gastric cancer development

Number of subjects who developed gastric cancer

	Basic model with *H. pylori* antibody			
Basic model	<4.0%	4.0%-10.0%	>10.0%	Total
<4.0%	22	6	0	28
4.0%-10.0%	2	23	8	33
>10.0%	0	5	57	62
Total	24	34	65	123

Number of subjects who did not develop gastric cancer

	Basic model with *H. pylori* antibody			
Basic model	<4.0%	4.0%-10.0%	>10.0%	Total
<4.0%	988	100	0	1,088
4.0%-10.0%	176	503	71	750
>10.0%	0	69	356	425
Total	1,164	672	427	2,263

H. pylori, *Helicobacter pylori*; NRI, net reclassification improvement.

The basic model included age, sex, body mass index, total cholesterol, hemoglobin A1c, smoking habits, and daily total energy and salt intakes.

The median value of the predicted probabilities by basic model with *H. pylori* antibody was 3.8% in subjects without occurrence of gastric cancer and 10.3% in those with gastric cancer.

Categorical NRI = 0.090, Z_{NRI} = 2.34, P = 0.02, continuous NRI = 0.330, Z_{NRI} = 3.56, P<0.001.
