# Supplementary material for: Combination of Helicobacter pylori Antibody and Serum Pepsinogen as a Good Predictive Tool of Gastric Cancer Incidence: 20-Year Prospective Data From the Hisayama Study
Source: J Epidemiol. 2016 Dec 5;26(12):629–36. doi: 10.2188/jea.JE20150258 (PMC5121431; doi:10.2188/jea.JE20150258)
Supplement: eTable 2. [file je-26-629-s002.pdf]

eTable 2. Reclassification for 20-year predicted absolute risk of gastric cancer development accounting for competing risk of death

Number of subjects who developed gastric cancer

Basic model with *H. pylori* antibody	Basic model with the combination of *H. pylori* antibody and sPG			
	<3.0%	3.0%-10.0%	>10.0%	Total
<3.0%	9	11	1	21
3.0%-10.0%	9	24	22	55
>10.0%	0	10	37	62
Total	18	45	60	123

Number of subjects who did not develop gastric cancer

Basic model with *H. pylori* antibody	Basic model with the combination of *H. pylori* antibody and sPG			
	<3.0%	3.0%-10.0%	>10.0%	Total
<3.0%	799	187	7	933
3.0%-10.0%	334	525	114	973
>10.0%	0	137	160	297
Total	1,133	849	281	2,263

H. pylori, *Helicobacter pylori*; sPG, serum pepsinogen; NRI, net reclassification improvement.
The basic model included age, sex, body mass index, total cholesterol, hemoglobin A1c, smoking habits, and daily total energy and salt intakes. In this analysis, the absolute risks for individuals were calculated by using Fine and Gray method for taking competing risk of death into account.
The median value of the predicted probabilities by basic model with the combination of *H. pylori* antibody and sPG was 3.0% in subjects without occurrence of gastric cancer and 9.5% in those with gastric cancer.
Categorical NRI = 0.194, Z_{NRI} = 3.21, P = 0.001, continuous NRI = 0.591, Z_{NRI} = 6.38, $P < 0.001$.
