# Supplementary material for: Combination of Helicobacter pylori Antibody and Serum Pepsinogen as a Good Predictive Tool of Gastric Cancer Incidence: 20-Year Prospective Data From the Hisayama Study
Source: J Epidemiol. 2016 Dec 5;26(12):629–36. doi: 10.2188/jea.JE20150258 (PMC5121431; doi:10.2188/jea.JE20150258)
Supplement: Abstract in Japanese. [file je-26-629-s003.pdf]

ヘリコバクター・ピロリ抗体・ペプシノゲン法の組み合わせは胃癌罹患の有用な予測手段である：久山町研究　20年間の追跡調査から

池田文恵[1,2,3]，志方健太郎[1,2]，秦　淳[1,2,3]，福原正代[1,2]，平川洋一郎[1,2]，小原知之[1,4]，向井直子[1,2,3]，永田雅治[1,2]，吉田大悟[1,3]，米本孝二[5]，江崎幹宏[2]，北園孝成[2,3]，清原　裕[1,3]，二宮利治[1,2,3]

[1]九州大学大学院医学研究院　環境医学分野
[2]九州大学大学院医学研究院　病態機能内科学
[3]九州大学大学院医学研究院　総合コホートセンター
[4]九州大学大学院医学研究院　精神病態医学
[5]久留米大学　バイオ統計センター

背景：萎縮性胃炎の程度の指標であるヘリコバクター・ピロリ（*Helicobacter pylori*：*H.pylori*）抗体価とペプシノゲン(sPG)検査を組み合わせた検査の、将来の胃癌罹患予測における有用性は、ほとんど検討されていない。そこで、わが国の地域住民を対象とした長期間の前向きコホート研究の成績を用いて、この問題について検討した。
方法：40歳以上の日本人地域住民2,446人を追跡開始時の*H.pylori*抗体陽性の有無とsPG検査の陽性の有無の組み合わせにより4群：A群(*H.pylori*[-]sPG[-])、B群(*H.pylori*[+]sPG[-])、C群(*H.pylori*[+]sPG[+])、D群(*H.pylori*[-]sPG[+])に分類し、前向きに20年間追跡した。
結果：追跡期間中に123人が胃癌を発症した。胃癌の累積罹患率は、A群に比べB、C、D群で有意に上昇したが、C群とD群の間に有意差は認められなかった。多変量調整後の胃癌罹患のハザード比は、B群　4.08(95%信頼区間1.62-10.28)、C+D群　11.1(95%信頼区間4.45-27.46)であった。*H.pylori*抗体価と胃癌の他の危険因子で構成された胃癌罹患の予測モデルを*H.pylori*抗体およびsPG検査の組み合わせと胃癌の他の危険因子で構成された予測モデルに変更したところ、予測モデルのC統計量は0.732から0.773と有意に上昇した（P=0.005）。また、Continuous net reclassification improvement(cNRI)も、2つの予測モデルの差が0.591と有意に胃癌罹患予測能は改善した（P<0.001）。
結論：*H.pylori*抗体価とsPG検査を組み合わせて用いることは、将来の胃癌罹患リスクを予測する上で有用な手段である。

Key words：ヘリコバクター・ピロリ、ペプシノゲン、前向き追跡研究、胃癌、生物学的指標
